# Royal Medical and Chirurgical Society of London

**Published:** 1888-03

**Authors:** 


### ROYAL MEDICAL AND CHIRURGICAL SOCIETY OF LONDON.

A very interesting case was shown by Dr. W. R. Gowers and Mr. Victor Horsley to the fellows of the society. It was that of a gentleman of middle age, who had for more than three years suffered with acute pain in about the middle of the back, just below and inside the lower angle of the left scapula. The spot was very tender as well as painful, but below the region of the fifth nerve there was almost complete anæsthesia as well as complete paraplegia; the accuracy of the delimitation was more distinct on the left side than the right. After some discussion the diagnosis of tumor of the spinal cord was agreed upon, and Mr. Victor Horsley undertook the operation necessary for its removal. On June 9, 1887, the spines and laminæ of the fifth and fourth dorsal vertebræ were removed without laying bare the cause of mischief ; but when the greater part of the posterior part of the third vertebra had been removed, a myxoma about the size of a filbert became visible in the spinal canal, lying on the right side, and compressing the spinal cord. This was shelled out without difficulty and no further new growth was found. The pain was for a time slightly relieved, but again and again recurred with great severity, and the power of motion was only slowly and intermittently regained during the first three or four weeks. The pressure over the wound was treated by a pad and strong jacket; the local pain gradually diminished; the motor power gradually returned to the lower parts; and after seven months the use of the lower limbs, though a little stiff, had become almost natural, and the remains of the laminæ had come so near together in the well-healed cicatrix that any further external support seemed unnecessary.

*Case of Intra-peritoneal Rupture of the Bladder; Abdominal Section; Suture of the Bladder; Recovery.* By W. J. WALSHAM, F. R. C. S.—C. H., aged 22, was admitted March 1, 1887, into St. Bartholomew's Hospital under the care of Mr. Walsham. He had been drinking the night before, and in a fight was butted by his opponent in the abdomen, his bladder being full at the time. He passed a night of great agony and was brought in a cab to the hospital the following morning, but he was then suffering very little shock, and walked into the surgery with the assistance of two friends. He complained of pain in the lower part of the abdomen, and of having been unable to pass any urine since the blow, although his bladder was uncomfortably full at the time. The perineum was natural, and there was no history of stricture. On passing a catheter no urine flowed, although the point was ascertained to be in the bladder by the finger in the rectum. On depressing the handle the catheter was felt to free itself with a jerk, and its point could be then felt more plainly than natural through the abdominal walls. Bloody urine now escaped, the flow varying with respiration. About twelve hours after the injury Mr. Walsham opened the abdomen, and having discovered an intra-peritoneal rent in the posterior wall of the bladder, sewed it up with nine Lembert sutures. The sutures were passed through the muscular and peritoneal coats only, and one was placed above and below the upper and lower angles of the wound respectively. The bladder having been forcibly injected with eight ounces of boric acid solution and found water tight, the peritoneum was irrigated with about two gallons of warm boric acid solution, and the abdominal wound closed as in ovariotomy. A catheter was left in the bladder for two hours, and the patient subsequently reminded to pass his urine every four hours. There was little shock and the patient recovered. Daily notes were given at length. The author remarked that there had now been seventeen cases in which abdominal section had been performed for rupture of the bladder, three extra-peritoneal and fourteen intra-peritoneal. Of the three extra-peritoneal cases two died and one recovered. In the successful case the wound in the bladder was secured to the abdominal wall but not sutured. In the fatal cases death was due to shock. The rent in one was found securely sutured at the *post-mortem* examination; in the other the rupture had not been discovered on opening the abdomen. Of the fourteen intra-peritoneal ruptures the rent in the bladder was sutured in eleven, and in three a drainage-tube was placed in the wound but no sutures employed. Of these three one recovered and two died, death being due to peritonitis and suppression of urine respectively, Of the eleven cases where the rent in the bladder was secured by sutures five recovered and six died, death being due in three cases to peritonitis, in two probably to shock, and in one to hæmorrhage from a perineal incision employed for exploration. In the three cases of peritonitis the sutures had given way in one, and a leakage had occurred in the lower part of the wound in the other two. In the five successful cases Lembert sutures were employed, and the peritoneum was washed out, and in only one was a drainage-tube used. The author discussed: 1, the advisability of early operation; 2, the importance of using a suture which will not become softened too soon, and of ascertaining before closing the abdominal wound that there is no leakage from the bladder; 3, the cleansing of the peritoneal cavity; 4, the inadvisability of a preliminary incision in the perineum, or of a subsequent incision in that region for the purpose of drain; and, 5, the question of tying in a catheter after the operation. A table of the seventeen cases was given, sixteen of which are in Sir William MacCormac's table appended to his work on "Abdominal Section."

Mr. HOLMES took a peculiar interest in the subject, and that, not only because he believed that he had been the first surgeon to suggest this method of treatment. It was a suggestion that must have become obvious as soon as we learnt how freely it was possible to deal with the abdomen. The preliminary diagnosis was sometimes very difficult. He had seen a case in which the *post-mortem* examination showed a rupture, but in which the patient during life had passed water without great difficulty. Dr. Weis, of Philadelphia, had suggested the preliminary injection of the bladder with fluid, but that, he thought, added little to the certainty in doubtful cases. The reason of the retention of the capacity to pass water after rupture was still to seek. If, as he was inclined to think, it might depend on the fact that the rent in the wall of the bladder was incomplete at first, then preliminary injection would be very dangerous. If it was due to the temporary plugging of the rent by the intestines, the safer course would be to cut down upon it early. It was important for diagnosis to make a preliminary exploration with a catheter. In his own case that had been sufficient to render the diagnosis quite plain. He doubted whether it was a good plan to inject the bladder after putting in the sutures; it might help to tear them out, and if they were near enough together it would be probably unnecessary. In all other points he cordially agreed with Mr. Walsham's treatment. Perineal incision had better be avoided; it was the cause of death in Mr. Teale's case, and had been merely vexatious in his own. He thought we should come to regard these cases as comparatively easy, so great was the improvement in abdominal surgery, compared to distant days, when he could remember seeing a patient with ruptured bladder but still looking fairly strong, come under Mr. Cæsar Hawkins's treatment, who diagnosed his injury rightly enough, but had nothing further to do than to watch him die of peritonitis.

Mr. BUTLIN said that he had made a *post-mortem* on a case of Mr. Willett's, in which an orifice had been found in a sutured bladder after death, and that had led several who had seen it to make up their minds not to refrain from injecting the bladder after suture in any similar case.

Mr. T. SMITH remarked that the operation could not be called completed until the bladder had been proved water-tight, as in cases of vesico-vaginal fistula.

Mr. BARWELL approved of the injections before and after operation, though he had had no opportunity of practicing them.

Mr. WALSHAM, in reply, had only a few words to say. He thought the preliminary injection might do a little good and no harm; and the injection subsequent to the sutures, he felt important, for in three fatal cases a leak had been shown to exist.

